# Genetic association mapping leveraging Gaussian processes

**DOI:** 10.1038/s10038-024-01259-0

**Published:** 2024-06-04

**Authors:** Natsuhiko Kumasaka

**Affiliations:** Division of Digital Genomics, Human Genome Center, Institute of Medical Science, The University of Tokyo, Tokyo, Japan

**Keywords:** Genome-wide association studies, Gene regulation

## Abstract

Gaussian processes (GPs) are a powerful and useful approach for modelling nonlinear phenomena in various scientific fields, including genomics and genetics. This review focuses on the application of GPs in genetic association mapping. The aim is to identify genetic variants that alter gene regulation along continuous cellular states at the molecular level, as well as disease susceptibility over time and space at the population level. The challenges and opportunities in this field are also addressed.

## Introduction

A genome-wide association study (GWAS) is a powerful approach for identifying genetic associations and related genes involved in the molecular mechanisms of common complex traits, such as diabetes and human height [1]. This approach was first introduced by a Japanese group in the study of myocardial infarction [2]. As of April 2023, the GWAS Catalog reports 496,341 genetic associations discovered through GWAS of 56,399 common complex traits [3].

In a GWAS, a phenotype *y* of a trait of interest is regressed onto a genotype *g* at a common genetic variant, usually a single nucleotide polymorphism (SNP), observed in a homogeneous population. If the phenotype *y* takes binary values, the logistic regression ${{{{{{{\rm{logit}}}}}}}}\,{\mathbb{E}}[y]=\alpha +\beta g$ is used. For a quantitative phenotype such as height or BMI, a typical linear regression model *y* = *α* + *βg* + *ε* is used. Here, {*α*, *β*} represent the coefficients of the (generalized) linear model and *ε* represents the residual. Here we assume an additive genetic model and a biallelic genetic variant *g* ∈ {0, 1, 2} representing the number of alternative alleles of a genotype at the variant. Statistical hypothesis testing is conducted for each of the tens of millions of genome-wide variants, with the null hypothesis being *β* = 0.

It is known that, the rationale of genetic association mapping is feasible not only for a common complex traits, but also for various cellular traits, such as gene expression and chromatin accessibility. For a quantitative cellular trait, it is referred to as a cellular quantitative trait locus (cellular QTL), and specifically, an eQTL (expression QTL) when the trait is related to gene expression [4]. The recent advances in sequencing technology allow for directly counting DNA and cDNA molecules to accurately quantify cellular traits. This has led to the development of new methods for modelling *y* using a discrete probability distribution, such as the Poission distribution or negative binomial distribution, with a mean $\log {\mathbb{E}}[y]=\alpha +\beta g$ denoting the additive effect of *g* on the $\log$-transformed phenotype. For instance [5], employed a combination of negative binomial and beta binomial distributions to model the cellular QTL effect for the total feature counts, as well as allele-specific feature counts at each active feature.

However, the linear models mentioned above assume the conditional independence, *i.e*.$p({y}_{1},\ldots ,{y}_{N}| \,{g}_{1},\ldots ,{g}_{N})=\prod \nolimits_{i = 1}^{N}p({y}_{i}| {g}_{i})$, of *N* observations ${\{{y}_{i},{g}_{i}\}}_{i = 1}^{N}$, which is only valid for unrelated samples and not for repeated samples from a single donor or correlated samples in general. When this assumption is violated, test statistic inflation occurs, leading to an increased false positive rate of genetic associations [6]. In addition, if the association effect size *β* varies along with an intrinsic or extrinsic factor *x*, the association mapping of such a dynamic genetic effect (*β* = *β*_*x*_) may often be underpowered [7–9].

This review provides an overview of robust and sensitive approaches to map a dynamic genetic effect *β*_*x*_ as a function of a continuous factor *x* within the framework of Gaussian Processes, in which an appropriate statistical calibration is also applied. As a case study, we also demonstrate a recent effort to map a dynamic eQTLs in single-cell resolution that manifest during the innate immune response in human fibroblasts [10].

## Association mapping leveraging Gaussian Processes

### Gaussian Process (GP)

Gaussian Process (GP) is a type of stochastic processes, whose application in the machine learning field enables us to infer a nonlinear function *f*(*x*) over a continuous domain *x* (*e.g*., time and space). Precisely, *f*(*x*) is a draw from a GP, if {*f*(*x*_1_), …, *f*(*x*_*N*_)} follows a *N*-dimensional multivariate normal distribution for the *N* input data points ${\{{x}_{i}\}}_{i = 1}^{N}$. Let us denote $X={({x}_{1},\ldots ,{x}_{N})}^{\top }$ and $f={(f({x}_{1}),\ldots ,f({x}_{N}))}^{\top }$, a GP is formally written as

$$f \sim {{{{{{{\mathcal{N}}}}}}}}(m(X),k(X,X)),$$

where *m*(⋅) denotes the mean function and *k*(⋅,⋅) denotes the kernel function [11]. The simplest kernel function would be the linear kernel, such that *k*(*X*, *X*) = *σ*^2^*XX*^⊤^, while the automatic relevance determination squared exponential (ARD-SE) kernel is defined as

$$k({x}_{j},{x}_{k})={\sigma }^{2}\exp \left[-{\sum }_{q=1}^{Q}\frac{{({x}_{jq}-{x}_{kq})}^{2}}{2{\rho }_{q}}\right]$$

for the (*j*, *k*) element of *k*(*X*, *X*), where ${x}_{j},{x}_{k}\in {{\mathbb{R}}}^{Q}$ are *Q*-dimensional input vectors. Here *σ*^2^ is the kernel variance parameter and $\rho ={({\rho }_{1},\ldots ,{\rho }_{Q})}^{\top }$ is the vector of characteristic length scales, whose inverse determines the relevance of each element of the input vector. Typically, the mean function is defined as *m*(*X*) = 0.

### GP regression and its use in genetic association mapping

Because the GP yielding *f*(*x*) has various useful properties inherited from the normal distribution, GP can be used to estimate a nonlinear function *f*(*X*) from output data $y={({y}_{1},\ldots ,{y}_{N})}^{\top }$ along continuous factor *X*. The extended linear model *y* = *f*(*X*) + *ε* is referred to as the GP regression and widely used in the machine learning framework [12]. This model can be used to map dynamic genetic associations for normalized gene expression or other common complex quantitative traits (*e.g*., human height) along the continuous factor *x* (*e.g*., cellular states or donor’s age). Let us denote the genotype vector $g={({g}_{1},\ldots ,{g}_{N})}^{\top }$ and the kinship matrix *R* among *N* individuals, the mapping model, as proposed by us or others [8, 10] can be expressed as follows:

1 $$y=\alpha +\beta \odot g+\gamma +\varepsilon ,$$

where

$$\alpha \sim {{{{{{{\mathcal{N}}}}}}}}(0,K),\quad \beta \sim {{{{{{{\mathcal{N}}}}}}}}(0,{\delta }_{g}K),\quad \gamma \sim {{{{{{{\mathcal{N}}}}}}}}(0,{\delta }_{d}K\odot R)$$

are all GPs with similar covariance matrices, where ⊙ denotes element wise product between two vectors or matrices with the same dimensions, *K* = *k*(*X*, *X*) denotes the covariance matrix with a kernel function, and *ε* denotes the residuals. Intuitively, *α* models the average baseline change of *y* in relation to *x*, while *β* represents the dynamic genetic effect along *x*. The effect size is multiplied by the genotype vector *g*, indicating that the output *y*_*i*_ varies between different genotype groups (*g*_*i*_ ∈ {0, 1, 2}). In fact, the effect size *β*(*x*_*i*_) is additive to the baseline *α*(*x*_*i*_) at each *x*_*i*_, which is the same as the standard association mapping. Here statistical hypothesis testing is performed under the null hypothesis of *δ*_*g*_ = 0, as the strength of genetic association is determined by *δ*_*g*_.

It is important to note that the model (1) includes a correction term *γ* that accounts for the between-donor variation of dynamic changes along *x*, particularly when multiple data points are measured from the same donor or samples are taken from related donors. This term is essential for statistical calibration of the genetic effect *β*, because other genetic associations scattered over the genome (trans effects) can confound the target genotype effect. Therefore, to adjust for the confounding effect, we need to include the extra GP *γ*, which is drawn from a normal distribution with the covariance matrix of *K* multiplied by the kinship matrix *R*.

Here, the kinship matrix is estimated by $\hat{R}=\sum\nolimits_{l = 1}^{L}{\tilde{g}}_{l}{\tilde{g}}_{l}^{\top }/L$ using genome-wide variants *g*_*l*_(*l* = 1, …,*L*), where ${\tilde{g}}_{l}$ is a standardized genotype vector (centered and scalced) based on the allele frequency at genetic variant *l*, while *L* denotes the total number of all variants across the genome [6]. The matrix is initially a *N* × *N* dense matrix, but it can be simplified if donors are (sufficiently) unrelated. Let us introduce a design matrix of donor configuration, $Z\in {{\mathbb{R}}}^{N\times {N}_{d}}$, for the *N*_*d*_ donors (*i.e*., *z*_*ij*_ = 1 if the sample *i* is taken from the donor *j*; otherwise *z*_*ij*_ = 0), the kinship matrix can then be approximated as *R* = *ZZ*^⊤^. Thus, *γ* can be expressed as a linear combination of *N*_*d*_ independent GPs $\{{\gamma }_{j} \sim {{{{{{{\mathcal{N}}}}}}}}(0,{\delta }_{d}K);j=1,\ldots ,{N}_{d}\}$, such that $\gamma =\mathop{\sum }\nolimits_{j = 1}^{{N}_{d}}{\gamma }_{j}\odot {z}_{j}$, where *z*_*j*_ denotes the *j*th column vector of *Z*. This approximation is particularly useful for parameter estimation with large *N*_*d*_ (as discussed in section 2.4).

### Sparse GP and Titsias bound

When the sample size *N* is large, an ordinary GP faces a severe scalability issue due to the dimension of the dense matrix *K* being *N* × *N*, resulting in a total computational cost of ${{{{{{{\mathcal{O}}}}}}}}({N}^{3})$. As a result, the application of GP in the GWAS field is hindered, as the sample sizes often reach a million these days. However, there are several alternatives to approximate the full GP model, including Nyström approximation (low-rank approximation), Projected Process approximation [13], Sparse Pseudo-inputs GP [14], Fully Independent Training Conditional approximation and Variational Free Energy approximation [15]. In this section, we introduce a sparse GP approximation proposed by [16].

The sparse GP is a scalable model using the technique of inducing points [14]. Since the computational cost of the sparse GP is ${{{{{{{\mathcal{O}}}}}}}}(N{M}^{2})$ with *M* inducing points, we can greatly reduce the computational cost, which is essentially linear to *N* under the assumption of *M* ≪ *N*. Let us denote *M* inducing points by $T={({t}_{1},\ldots ,{t}_{M})}^{\top }$ and corresponding GPs by $u={(u({t}_{1}),\ldots ,u({t}_{M}))}^{\top }$, the joint distribution of *f* and *u* becomes a multivariate normal distribution. Therefore a lower bound of the conditional distribution *p*(*y*∣*u*) can be written as

$$\log p(y| u) 	= \log \int\,p(y| f)p(f| u)df\ge \int\left[\log p(y| f)\right]p(f| u)df\\ 	= \log {{{{{{{\mathcal{N}}}}}}}}(y| \bar{f},{\sigma }^{2}I)-\frac{1}{2{\sigma }^{2}}{{{{{{{\rm{tr}}}}}}}}\{{\tilde{K}}_{NN}\}\equiv {{{{{{{{\mathcal{L}}}}}}}}}_{1},$$

where

$$\bar{f}={K}_{NM}{K}_{MM}^{-1}u,\quad {\tilde{K}}_{NN}={K}_{NN}-{K}_{NM}{K}_{MM}^{-1}{K}_{MN},$$

and

$${K}_{NN}=k(X,X),\quad {K}_{NM}=k(X,T),\quad {K}_{MM}=k(T,T).$$

Therefore, the marginal distribution of the output *y* is approximated by

$$p(y) 	 = \int\,p(y| u)p(u)du\ge \int\exp \{{{{{{{{{\mathcal{L}}}}}}}}}_{1}\}p(u)du\\ 	 = \log {{{{{{{\mathcal{N}}}}}}}}(y| 0,V)-\frac{1}{2{\sigma }^{2}}{{{{{{{\rm{tr}}}}}}}}\{{\tilde{K}}_{NN}\}\equiv \exp \{{{{{{{{{\mathcal{L}}}}}}}}}_{2}\},$$

where $V={\sigma }^{2}I+{K}_{NM}{K}_{MM}^{-1}{K}_{MN}$. The lower bound ${{{{{{{{\mathcal{L}}}}}}}}}_{2}$ is referred to as the *Titsias bound* and can be used for parameter estimation as well as statistical hypothesis testing.

Selecting the optimal number of inducing points *M* and their coordinates is crucial for accurately approximating a GP. Although a larger value of *M* provides a better approximation of GP, it is not feasible to increase *M* when *N* reaches hundreds of thousands in large-scale genetic association studies. Additionally, the accuracy of the GP is influenced by the complexity of nonlinearity of *y* and the dimension *Q* of input points *x*. There are few approaches inferring an optimal value of *M* from data [17], but the size of the example used in the study is too small (48 genes × 437 samples) to be applied to real-world data. However, it is worth noting that the optimal coordinate of inducing points with a fixed *M* can be easily learned from data, as described in the next section.

### Parameter estimation

Genetic association mapping involves performing tens of millions of hypothesis tests. Therefore, it is almost impossible to estimate the parameters of GPs from each pair of trait and variant across the genome, even with use of the sparse approximation mentioned in the last subsection. Furthermore, both the baseline *α* and the correction term *γ* share the characteristic length parameter $\rho ={({\rho }_{1},\ldots ,{\rho }_{Q})}^{\top }$ and the inducing points *T*. This can lead to unstable optimization and prolonged parameter estimation times. To address this issue, we have previously proposed a three-step parameter estimation strategy for performing the statistical hypothesis testing [10]. Especially, optimizing with respect to *α* using a quasi-Newton approach (such as the BFGS method) is sufficient in the first step, because the variance explained by *γ* is typically much smaller than that explained by *α*. The three steps are:

1. *y* = *α* + *ε* (baseline model: *H*_0_) to estimate *ρ* and *T*.
2. *y* = *α* + *γ* + *ε* (baseline model: *H*_1_) to estimate variance parameters *δ*_*d*_ and *σ*^2^. Here $\hat{\rho }$ and $\hat{T}$ estimated in *H*_0_ are plugged into *H*_1_.
3. *y* = *α* + *β* ⊙ *g* + *γ* + *ε* (full model: *H*_2_) to test whether *δ*_*g*_ = 0. Here $\{\hat{\rho },\hat{T},{\hat{\delta }}_{d},{\hat{\sigma }}^{2}\}$ estimated in *H*_0_ and *H*_1_ are used.

Here the Titsias bounds for these models are given by

$${{{{{{{{\mathcal{L}}}}}}}}}_{2}^{h}=\left\{\begin{array}{ll}\log {{{{{{{\mathcal{N}}}}}}}}(y| 0,V)-\frac{1}{2{\sigma }^{2}}{{{{{{{\rm{tr}}}}}}}}\{{\tilde{K}}_{NN}\},\hfill &h={H}_{0},\\ \log {{{{{{{\mathcal{N}}}}}}}}(y| 0,{V}_{d})-\frac{1}{2{\sigma }^{2}}{{{{{{{\rm{tr}}}}}}}}\{(1+{\delta }_{d}){\tilde{K}}_{NN}\},\hfill&h={H}_{1},\\ \log {{{{{{{\mathcal{N}}}}}}}}(y| 0,{V}_{g})-\frac{1}{2{\sigma }^{2}}{{{{{{{\rm{tr}}}}}}}}\{(1+{\delta }_{d}){\tilde{K}}_{NN}+{\delta }_{g}G{\tilde{K}}_{NN}G\},&h={H}_{2},\end{array}\right.$$

where

$${V}_{d}=V+{\delta }_{d}({K}_{NM}{K}_{MM}^{-1}{K}_{MN})\odot R,\quad {V}_{g}={V}_{d}+{\delta }_{g}G{K}_{NM}{K}_{MM}^{-1}{K}_{MN}G,$$

and *G* = diag(*g*) denotes the diagonal matrix whose diagonal elements are given by the elements of *g*. The estimators $\hat{\rho }$ and $\hat{T}$ are obtained by maximizing ${{{{{{{{\mathcal{L}}}}}}}}}_{2}^{{H}_{0}}$ with respect to *ρ* and *T*, and ${\hat{\delta }}_{d}$ and ${\hat{\sigma }}^{2}$ are obtained by maximizing ${{{{{{{{\mathcal{L}}}}}}}}}_{2}^{{H}_{1}}$ with respect to *δ*_*d*_ and *σ*^2^ given $\hat{\rho }$ and $\hat{T}$.

It is worth noting that, when the kinship matrix *R* can be expressed as *R* = *ZZ*^⊤^ with a lower rank matrix $Z=({z}_{1},\ldots ,{z}_{{N}_{d}})$ with *N*_*d*_ < *N*, *V*_*d*_ can be rewritten as

$${V}_{d}=V+{\delta }_{d}\left({K}_{NM}{K}_{MM}^{-1}{K}_{MN}\right)\odot (Z{Z}^{\top })={\sigma }^{2}I+A{B}^{-1}{A}^{\top },$$

where

$$A = 	\, (C,{{{{{{{\rm{diag}}}}}}}}({z}_{1})C,\ldots ,{{{{{{{\rm{diag}}}}}}}}({z}_{D})C),\quad \\ B = 	\, {{{{{{{\rm{diag}}}}}}}}({K}_{MM},{\delta }_{d}{K}_{MM},\ldots ,{\delta }_{d}{K}_{MM}),$$

and $C={K}_{NM}{K}_{MM}^{-1}$, and *B* becomes a *M*(*N*_*d*_ + 1) × *M*(*N*_*d*_ + 1) block diagonal matrix. Since the computational complexity of *H*_1_ or *H*_2_ is ${{{{{{{\mathcal{O}}}}}}}}({N}_{d}^{2}{M}^{2}N)$, for large *N*_*d*_ such as *MN*_*d*_ > *N*, the total complexity is over ${{{{{{{\mathcal{O}}}}}}}}({N}^{3})$ and we again face the scalability issue.

However, if the donors in the data are unrelated, we can significantly reduce the memory usage and the computational burden to be ${{{{{{{\mathcal{O}}}}}}}}({N}_{d}{M}^{2}N)$. This is because the matrix *A* becomes a sparse matrix, with ${z}_{i}^{\top }{z}_{{i}^{{\prime} }}=0$ for $i\ne {i}^{{\prime} }$, resulting in *NM*(*N*_*d*_ − 1) elements out of *NMN*_*d*_ bing 0. Additionaly, non-zero elements of *A* are repeated and identical to the elements of *C*, and the block diagonal element of *B* is essentially ${K}_{MM}^{-1}$.

### Score statistic and Bayes factor for genetic mapping

To perform GWAS with GP, it is crucial to reduce the computational time required to map a genetic association for each variant. The Score statistic to test *δ*_*g*_ = 0 can be computed from the first derivative of ${{{{{{{{\mathcal{L}}}}}}}}}_{2}^{{H}_{2}}$ with respect to *δ*_*g*_, and the variance parameters $\{{\hat{\sigma }}^{2},{\hat{\delta }}_{d}\}$ of *V*_*d*_ are estimated from ${{{{{{{{\mathcal{L}}}}}}}}}_{2}^{{H}_{1}}$ once for every single variant to be tested. Therefore, it is ideal to test tens of millions of variants independently. To use the fact that the first derivative of ${V}_{g}^{-1}$ given *δ*_*g*_ = 0 depends only on *V*_*d*_, such that

$${\left.\frac{\partial {V}_{g}^{-1}}{\partial {\delta }_{g}}\right\vert }_{{\delta }_{g} = 0}=-{V}_{d}^{-1}G{K}_{NM}{K}_{MM}^{-1}{K}_{MN}G{V}_{d}^{-1},$$

the Score statistic can be explicitly written as

2 $$S={y}^{\top }{\hat{V}}_{d}^{-1}G{K}_{NM}{K}_{MM}^{-1}{K}_{MN}G{\hat{V}}_{d}^{-1}y,$$

whose distribution is the generalized *χ*^2^ distribution, that is, the distribution of the weighted sum of *M* independent *χ*^2^ statistics, such as $\mathop{\sum }\nolimits_{m = 1}^{M}{\lambda }_{m}{\chi }_{m}^{2}$ [8, 10]. It is known that the weights *λ*_*m*_(*m* = 1, …, *M*) are given by the non-negative eigenvalues of

$${K}_{MM}^{-1/2}{K}_{MN}G{\hat{V}}_{d}^{-1}G{K}_{NM}{K}_{MM}^{-\top /2},$$

where ${K}_{MM}^{-1/2}$ can be computed using the Cholesky decomposition of ${K}_{MM}={K}_{MM}^{\top /2}{K}_{MM}^{1/2}$.

To compute the *p*-value from *S*, we can use the Davies’ exact method, implemented in the CompQuadForm package on R. Note that, if we use a linear kernel, *S* can be simplified as described [8]. Although the Score based approach is an easy and quick solution for genome-wide mapping, to check the asymptotic behavior and the statistical calibration of the Score statistics, we should use a QQ-plot to verify that the *p*-values obtained from multiple variants follow a uniform distribution under the null hypothesis.

If the collocalisation analysis [18] or Bayesian hierarchical model [19] is considered as a downstream analysis using the test statistics, a Bayes factor can also be computed using the Titsias bounds, such as

$$\log (BF)={{{{{{{{\mathcal{L}}}}}}}}}_{2}^{{H}_{2}}-{{{{{{{{\mathcal{L}}}}}}}}}_{2}^{{H}_{1}}.$$

Here we would use some empirical values *δ*_*g*_ = {0.01, 0.1, 0.5} to average the Bayes factor, instead of integrating out *δ*_*g*_ from ${{{{{{{{\mathcal{L}}}}}}}}}_{2}^{{H}_{2}}$ [20].

### Decomposing static and dynamic genetic associations

In a real genetic association mapping, most of genetic associations are indeed static and ubiquitous over the factor *x*. To capture such a static association, we can come up with the following model

$$y={\alpha }_{0}{1}_{N}+\alpha +{\beta }_{0}g+\beta \odot g+{\gamma }_{0}+\gamma +\varepsilon ,$$

where *α*_0_ denotes the intercept, 1_*N*_ denotes the *N*-dimensional vector of all 1’s, *β*_0_ denotes the effect size of the static genetic association, and ${\gamma }_{0} \sim {{{{{{{\mathcal{N}}}}}}}}(0,{\sigma }^{2}{\delta }_{d0}R)$ denotes the donor variation which confounds *β*_0_. For instance, in [8], the static genetic association *β*_0_ is modeled as a fixed effect, and the dynamic effect is tested using the Score statistic. On the other hand, in [10], the authors modeled both the static and dynamic associations as a random effect to test via a Bayes factor. In this case, the covariance matrix *K* can be rewritten as

$${K}^{* }={\sigma }^{2}{e}^{-{\rho }_{0}}{1}_{N}{1}_{N}^{\top }+K$$

to estimate the model parameters in (1), and then the variance *δ*_*g*_ = 0 for *β* is tested.

Note here that, the kernel parameter *ρ*_0_ is not necessarily common and shared across *α*, *β* and *γ*. Indeed, in [10], the authors estimated ${\hat{\rho }}_{0}^{\alpha }$ and ${\hat{\rho }}_{0}^{\gamma }$ independently in ${{{{{{{{\mathcal{L}}}}}}}}}_{2}^{{H}_{1}}$. To compute the Score statistic, the authors assumed that ${\hat{\rho }}_{0}^{\beta }={\hat{\rho }}_{0}^{\gamma }$ for *β* and *γ*, because the ratio of the static effect to the dynamic effect can be the same for cis and trans genetic effects.

### Latent variable *X*

In longitudinal studies, the factor *x* is typically observed explicitly (*e.g*., donor’s age or physical locations where samples were taken). This makes it straightforward to perform genetic association mapping along *x* using the Score statistics or Bayes factors, as described above. However, this is not often the case for the molecular studies, and therefore we need to estimate the underlying biological state from the data.

In single-cell biology, typically, the hidden cellular state *x* is often referred to as “pseudotime", and the principal component analysis is normally used to estimate it as part of dimension reduction [21]. Gaussian process latent variable model (GPLVM) is a strong alternative to extract the pseudotime when the molecular phenotype gradually changes along pseudotime *x* in a nonlinear fashion [22, 23].

We have also proposed a GPLVM that uses the baseline model *H*_0_ to estimate the latent variable *X* from the single-cell RNA-seq data (see Section 3 for more details). Let *Y* = (*y*_1_,…,*y*_*J*_) be the gene expression matrix of *J* genes, whose column is a vector of gene expression for the gene *j*, the Titsias lower bound of the GPLVM based on the baseline model *H*_0_ can be written as

$$p(Y| X)\ge {{{{{{{\mathcal{MN}}}}}}}}(Y| 0,\Sigma ,I+{K}_{NM}{K}_{MM}^{-1}{K}_{MN})-\frac{J}{2}{{{{{{{\rm{tr}}}}}}}}\{{\tilde{K}}_{NN}\}={{{{{{{{\mathcal{L}}}}}}}}}_{2}.$$

To obtain the optimal cellular state $\hat{X}$, this lower bound can be maximized with respect to {Σ, *X*, *T*, *ρ*} [10, 24]. Here $\Sigma ={{{{{{{\rm{diag}}}}}}}}({\sigma }_{j}^{2};j=1,\ldots ,J)$ denotes the residual variance parameters of *J* genes, and ${{{{{{{\mathcal{MN}}}}}}}}(\cdot )$ denotes the matrix normal distribution. Due to the uniqueness of the model parameters, the variance parameter in the kernel function is set to be *σ*^2^ = 1. In addition, to maintain the uniqueness of the latent variable estimation, a prior probability on *X* is required. It is quite common to assume independent standard normal distributions for each of the elements of $X \sim {{{{{{{\mathcal{MN}}}}}}}}(0,I,I)$ [24], although there are multiple alternatives to consider depending on the nature of the modeled data [10, 23].

In the parameter estimation, the limited-memory BFGS method can be used to implement GPLVM for large *N*. In addition, the stochastic variational Bayes approach can be used to fit GPLVM to larger data sets, while reducing the fitting time [25–27].

### Non Gaussian output *y*

For the non-Gaussian output *y*, the Titsias bound ${{{{{{{{\mathcal{L}}}}}}}}}_{2}$ is not analytically available. However, for the Poisson distribution case, a lower bound of the conditional probability *p*(*y*∣*u*) can be computed as follows:

$${{{{{{{{\mathcal{L}}}}}}}}}_{1}=\mathop{\sum}_{i}\left[-\log ({y}_{i}!)+{y}_{i}{\bar{f}}_{i}-\exp \left({\bar{f}}_{i}+\frac{{\tilde{k}}_{ii}}{2}\right)\right],$$

where ${\tilde{k}}_{ii}$ denotes the *i*th diagonal element of ${\tilde{K}}_{NN}$. Let *ν*_*i*_ and *w*_*i*_ be the working response and the iterative weight of GLM for the *i*th sample, such that

$${\nu }_{i}={\bar{f}}_{i}+({y}_{i}-{w}_{i})/{w}_{i}\quad {{{{{{{\rm{and}}}}}}}}\quad {w}_{i}=\exp \left({\bar{f}}_{i}+\frac{{\tilde{k}}_{ii}}{2}\right)$$

for *i* = 1, …, *N*, the optimal $\hat{u}$ which maximizes $\exp \{{{{{{{{{\mathcal{L}}}}}}}}}_{1}\}p(u)$ satisfies

3 $$\left({K}_{MM}^{-1}+{K}_{MM}^{-1}{K}_{MN}W{K}_{NM}{K}_{MM}^{-1}\right)u=W\nu ,$$

where *W* = diag(*w*_*i*_; *i* = 1, …, *N*), which suggests

$$\nu | u \sim {{{{{{{\mathcal{N}}}}}}}}(\bar{f},{W}^{-1})$$

as described in elsewhere [28]. Therefore, we can maximize

$${{{{{{{{\mathcal{L}}}}}}}}}_{2}={{{{{{{\mathcal{N}}}}}}}}(\nu | 0,{W}^{-1}+{K}_{NM}{K}_{MM}^{-1}{K}_{MN})$$

with respect to {*σ*^2^, *ρ*} where $u=\hat{u}$ is iteratively updated as in (3). Thus, to obtain the Score statistic for non-Gaussian *y*, we replace *y* = *ν* and ${\hat{V}}_{d}={W}^{-1}+A{B}^{-1}A$ in (2).

For a binary output *y*, it is more complicated than the Poisson case, bacause it is even impossible to analytically compute the ${{{{{{{{\mathcal{L}}}}}}}}}_{1}$ bound with logit or Probit link function. For logit link function, several useful alternatives to the ${{{{{{{{\mathcal{L}}}}}}}}}_{1}$ bound have been proposed [29]. For Probit link function [30], proposed an approximation of ${{{{{{{{\mathcal{L}}}}}}}}}_{1}$ using the Gauss-Hermite quadrature. However, in both cases, the computational cost is much higher than the Poisson case and it is rather impractical to conduct a large genome-wide association mapping at this moment.

## An application of GP in eQTL mapping

**Fig. 1:**
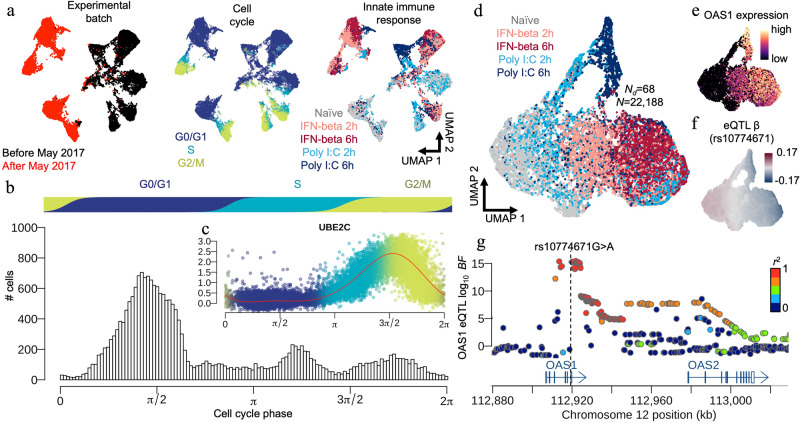
Single-cell analysis of innate immunity and OAS1 eQTL mapping in human skin fibroblasts. **a** Principal component analysis of the single cell RNA-seq data and its UMAP (Uniform Manifold Approximation and Projection) representation. Points are coloured by experimental batch (left), cell cycle (middle), and innate immune response for the two different cell stimuli at different time points (IFN-beta: Interferon-beta; Poly I:C: double-stranded RNA; Naïve: cell with no stimulation; IFN-beta 2h: cells stimulated with IFN-beta for 2 hours; IFN-beta 6h: cells stimulated with IFN-beta for 6 hours; Poly I:C 2h: cells stimulated with Poly I:C for 2 hours; Poly I:C 6h: cells stimulated with Poly I:C for 6 hours). **b** Latent cell cycle phase inferred by the periodic kernel implemented in our GPLVM. **c** An example gene (UBE2C) showing a cell cycle dependent expression pattern. **d** UMAP of Latent variable *X* estimated by our GPLVM representing innate immune responses with different cell stimuli. **e** UMAP showing gene expression level of OAS1. **f** UMAP showing OAS1 eQTL effect size *β* at rs10774671. **g** Regional plot showing the association Bayes factors (in ${\log }_{10}$ scale) of OAS1 eQTL around the OAS locus. One of the SNPs with the high Bayes factors is also a risk SNP of COVID-19

The authors proposed a novel GPLVM to extract innate immune responses in human skin fibroblasts at single cell resolution [10]. The baseline model (*H*_0_),

$${y}_{j}={\alpha }_{j}+W{b}_{j}+{\varepsilon }_{j}\quad (j=1,\ldots ,J),$$

involves covariates *W* (a design matrix of donors and experimental batches) to adjust for both intrinsic and extrinsic biases (Fig. 1a) embedded in the single-cell RNA-seq data. Here the effect ${b}_{j} \sim {{{{{{{\mathcal{N}}}}}}}}(0,{\sigma }_{j}^{2}\Delta )$ is a random effect and integrated out from the Titsias bound along with the GP *u*_*j*_ (on inducing points), such as

$${{{{{{{{\mathcal{L}}}}}}}}}_{2}={{{{{{{\mathcal{MN}}}}}}}}\left(Y| 0,\Sigma ,I+{K}_{NM}{K}_{MM}^{-1}{K}_{MN}+W\Delta {W}^{\top }\right)-\frac{J}{2}{{{{{{{\rm{tr}}}}}}}}\{{\tilde{K}}_{NN}\}.$$

In addition, we employed a combination of ARD-SE and periodic kernels

$$k({x}_{j},{x}_{k})=\exp \left[-\frac{{\sin }^{2}(({x}_{j1}-{x}_{k1})/2)}{{\rho }_{1}}\right]\exp \left[-\mathop{\sum }_{q=2}^{Q}\frac{{({x}_{jq}-{x}_{kq})}^{2}}{2{\rho }_{q}}\right]$$

to model the latent cell cycle phase (*x*_11_,…,*x*_*N*1_) from data to correct for the intrinsic biological bias (Fig. 1b, c).

We then performed a genetic association mapping along the extracted trajectories of innate immunity (Fig. 1d) and discovered the OAS1 eQTL which manifested through the secondary response of innate immunity (Fig. 1e, f). A subsequent colocalisation analysis with various immune-related GWAS traits demonstrated that the dynamic eQTL is colocalized with the OAS locus (Fig. 1g) identified by GWAS of COVID-19 susceptibility and severity [31]. For more details, readers may refer to [10].

## Discussion

This review presents an overview of (1) fundamental properties of GPs, (2) an approximation for scalable computations, and (3) a comprehensive model for genetic association mapping. We have also provided an example of single-cell eQTL mapping to demonstrate the power to detect context-specific dynamic genetic associations that arise during innate immune responses. Despite the fact that GPs are flexible and sensitive to nonlinear phenomena, they are explicitly described as a probabilistic model (essentially just a multivariate normal distribution), unlike neural networks or other deep learning approaches. This feature is very useful for understanding the underlying mechanisms of gene regulation and disease biology. Using GPLVM has the additional benefit of estimating latent variables, which can reveal hidden biological states behind the data. This is not only useful for genetic association mapping but also for other biological areas.

Compared to deep learning approaches, however, GP-based approaches are not well-known or appreciated in the field of genetics and genomics. This is likely due to the fact that explicitly described models are challenging to implement or use as a black box without mathematical knowledge and programming skills. Currently, there are only a few computational packages and environments available for implementing GP-based models in general (*e.g*., https://gpy.readthedocs.io/en/deploy/).

It is also true that GP faces a scalability issues when dealing with a large number of donors (*N*_*d*_) in genetic association mapping. As previously mentioned, the computational cost increases quadratically with in *N*_*d*_, making it difficult to apply GP to modern genetic data from millions of donors. However, if the donors are unrelated, there is a solution that significantly reduces the memory usage and computational time. This is because the design matrices used in the model become redundant and very sparse. Furthermore, the use of Score statistics instead of likelihood ratios or Bayes factors eliminates the need from millions of parameter estimation procedures and enables scalable association mapping.

It is worth mentioning that Random Fourier Features (RFF) can be a better alternative to sparse GP, also for large data [32]. It is now becoming popular in the field of machine learning, allowing GP to be fitted without the lower bound with less computational complexity. It is also extended to estimate the hidden state *x* using the RFF latent variable model [33].

The decrease in sequencing and genotyping costs has made it possible to conduct GWAS with millions of donors or eQTL mapping with tens of thousands of samples. However, computational methods for mapping nonlinear and dynamic genetic associations have not kept pace with the growth of such data. In addition, it is crucial to handle non-Gaussian output *y* for different data types and scales in the age of multimodal data analysis. This review aimed to explore the potential of dynamic genetic association mapping using GP for the future of more complex and comprehensive genetic studies.

## References

[1] Wellcome Trust Case Control Consortium. Genome-wide association study of 14,000 cases of seven common diseases and 3000 shared controls. Nature. 2007;447:661–78. 17554300 10.1038/nature05911 PMC2719288

[2] Ozaki K, Ohnishi Y, Iida A, Sekine A, Yamada R, Tsunoda T et al. Functional SNPs in the lymphotoxin-alpha gene that are associated with susceptibility to myocardial infarction. Nat Genet. 2002;32:650–54. 12426569 10.1038/ng1047

[3] Sollis E, Mosaku A, Abid A, Buniello A, Cerezo A, Gil L. et al. The NHGRI-EBI GWA S catalog: Knowledgebase and deposition resource. Nucleic Acids Res 2023;51:D977–85. 36350656 10.1093/nar/gkac1010 PMC9825413

[4] GTEx Consortium. The GTEx consortium atlas of genetic regulatory effects across human tissues. Science. 2020;369:1318–30. 32913098 10.1126/science.aaz1776 PMC7737656

[5] Kumasaka N, Knights AJ, Gaffney DJ. Fine-mapping cellular QTLs with RASQUAL and ATAC-seq. Nat Genet. 2016;48:206–13. 26656845 10.1038/ng.3467 PMC5098600

[6] Loh P-R, Tucker G, Bulik-Sullivan BK, Vilhjálmsson BJ, Finucane HK, Salem RM et al. Efficient Bayesian mixed-model analysis increases association power in large cohorts. Nat Genet. 2015;47:284–90. 25642633 10.1038/ng.3190 PMC4342297

[7] Moore R, Casale FP, Jan Bonder M, Horta D, Franke L.BIOS Consortium et al. A linear mixed-model approach to study multivariate gene-environment interactions. Nat Genet. 2019;51:180–86. 10.1038/s41588-018-0271-0 PMC6354905 30478441

[8] Cuomo ASE, Heinen T, Vagiaki D, Horta D, Marioni JC, Stegle O. CellRegMap: a statistical framework for mapping context-specific regulatory variants using scRNA-seq. Mol Syst Biol2022;18:e10663. 35972065 10.15252/msb.202110663 PMC9380406

[9] Cuomo, ASE, Nathan, A, Raychaudhuri, S, MacArthur, DG & Powell, JE. Single-cell genomics meets human genetics. Nat Rev Genet. 2023;24:535–49 10.1038/s41576-023-00599-5 PMC10784789 37085594

[10] Kumasaka N, Rostom R, Huang N, Polanski K, Meyer KB, Patel S et al. Mapping interindividual dynamics of innate immune response at single-cell resolution. Nat Genet. 2023;55:1066–75. 37308670 10.1038/s41588-023-01421-y PMC10260404

[11] MacKay, DJC.*Information Theory, Inference, and Learning Algorithms* (Copyright Cambridge University Press, 2003).

[12] Rasmussen, CE & Williams, CKI. *Gaussian processes for machine learning* (MIT Press, 2005).

[13] Seeger, M *Bayesian Gaussian process models: PAC-Bayesian generalisation error bounds and sparse approximations* (PhD thesis, University of Edinburgh, 2003).

[14] Snelson, E & Ghahramani, Z. Sparse gaussian processes using pseudo-inputs. In Weiss, Y., Schölkopf, B. & Platt, J.C. (eds.) *Advances in Neural Information Processing Systems 18*, 1257–1264 (MIT Press, 2006).

[15] Bauer, M, van der Wilk, M & Rasmussen, CE. Understanding probabilistic sparse gaussian process approximations. Adv Neural Inf Process Syst. 2016;29:1533–41.

[16] Titsias, M. Variational learning of inducing variables in sparse Gaussian processes. Proceedings of the Twelfth International Conference on Artificial Intelligence and Statistics. PMLR. 2009;5:567–74.

[17] Uhrenholt, AK, Charvet, V & Jensen, BS. Probabilistic selection of inducing points in sparse gaussian processes. In de Campos, C. & Maathuis, M.H. (eds.) *Proceedings of the Thirty-Seventh Conference on Uncertainty in Artificial Intelligence*, vol. 161 of *Proceedings of Machine Learning Research*, 1035–1044 (PMLR, 2021).

[18] Giambartolomei C, Vukcevic D, Schadt EE, Franke L, Hingorani AD, Wallace C et al. Bayesian test for colocalisation between pairs of genetic association studies using summary statistics. PLoS Genet. 2014;10:e1004383. 24830394 10.1371/journal.pgen.1004383 PMC4022491

[19] Veyrieras J-B, Kudaravalli S, Kim Su Y, Dermitzakis ET, Gilad Y, Stephens M et al. High-resolution mapping of expression-QTLs yields insight into human gene regulation. PLoS Genet 2008;4:e1000214. 18846210 10.1371/journal.pgen.1000214 PMC2556086

[20] Pickrell JK, Berisa T, Liu JZ, Ségurel L, Tung JY, Hinds DA Detection and interpretation of shared genetic influences on 42 human traits. Nat Genet. 2016;48:709–17. 27182965 10.1038/ng.3570 PMC5207801

[21] Haghverdi L, Büttner M, Wolf FA, Buettner F, Theis FJ. Diffusion pseudotime robustly reconstructs lineage branching. Nat Methods. 2016;13:845–48. 27571553 10.1038/nmeth.3971

[22] Campbell KR, Yau C. Order under uncertainty: Robust differential expression analysis using probabilistic models for pseudotime inference. PLoS Comput Biol. 2016;12:e1005212. 27870852 10.1371/journal.pcbi.1005212 PMC5117567

[23] Ahmed S, Rattray M, Boukouvalas A. GrandPrix: scaling up the bayesian GPLVM for single-cell data. Bioinformatics. 2019;35:47–54. 30561544 10.1093/bioinformatics/bty533 PMC6298059

[24] Lawrence N. Probabilistic non-linear principal component analysis with gaussian process latent variable models. J Mach Learn Res. 2005;6:1783–1816.

[25] Titsias M, Lawrence ND. Bayesian Gaussian process latent variable model. Proceedings of the Thirteenth International Conference on Artificial Intelligence and Statistics. PMLR. 2010;9:844–51.

[26] Hensman J, Fusi N, Lawrence ND. Gaussian processes for Big Data. arXiv:1309.6835 [Preprint]. 2013 [cited 2013 Sep 26]: [9 p.]. Available from: http://arxiv.org/abs/1309.6835.

[27] Lalchand V, Ravuri A, Dann E, Kumasaka N, Sumanaweera D, Lindeboom RGH et al. Modelling technical and biological effects in scRNA-seq data with scalable GPLVMs. PMLR. 2022;200:46–60.

[28] Breslow NE, Clayton DG. Approximate inference in generalized linear mixed models. J Am Stat Assoc. 1993;88:9–25.

[29] Marlin, BM, Khan, ME & Murphy, KP. Piecewise bounds for estimating bernoulli-logistic latent gaussian models. *Proceedings of the 28th International Conference on Machine Learning* (2011).

[30] Hensman J, Matthews A, Ghahramani Z. Scalable variational Gaussian process classification. Proc 18th Int Conf Artif Intell Stat. 2015;38:351–60.

[31] COVID-19 Host Genetics Initiative. Mapping the human genetic architecture of COVID-19. Nature. 2021;600:472–77. 34237774 10.1038/s41586-021-03767-x PMC8674144

[32] Rahimi, A & Recht, B. Random features for large-scale kernel machines. Adv. Neural Inf. Process. Syst. 2007;20:1177–84.

[33] Gundersen G, Zhang M, Engelhardt B. Latent variable modeling with random features. Proceedings of the 24th International Conference on Artificial Intelligence and Statistics. PMLR. 2021;130:1333–41.

